# Social anxiety mediates between victimization experiences and internet addiction among adolescents: results from propensity score matching

**DOI:** 10.3389/fpsyg.2024.1378428

**Published:** 2024-05-27

**Authors:** Jianwei Wu, Hui Wang, Xiang Li, Iat Kio Van, Xuebin Xie, Ching Han Pang

**Affiliations:** ^1^Kiang Wu Nursing College of Macau, Complexo de Cuidados de Saúde das Ilhas – Edifício do Instituto de Enfermagem Kiang Wu de Macau, Avenida do Hospital das Ilhas no. 447, Macao, Macao SAR, China; ^2^Kiang Wu Hospital, Macau, Macao SAR, China

**Keywords:** social anxiety, victimization experiences, internet addiction, adolescents, propensity score matching

## Abstract

**Background:**

Previous research has indicated that Victimization Experiences (VE) may be linked to a heightened likelihood of developing psychological symptoms and Internet Addiction (IA) among adolescents. However, the precise mechanism through which VE contributes to IA in adolescents remains uncertain. This study aimed to investigate whether Social Anxiety (SA) serves as a mediation between VE and IA, utilizing the framework of General Strain Theory.

**Methods:**

A cross-sectional survey among 11 middle schools or high schools in Macao was conducted from October to December 2022. Respondents in the victimized group and non-victimized group were 1:1 paired using Propensity Score Matching (PSM) to control the potential confounding factors.

**Results:**

A total of 1,089 questionnaires were valid for analysis and 311 pairs were generated through PSM. Respondents in the victimized group reported significantly higher IA than those in non-victimized group (23.5% vs. 12.5%, *p* < 0.001) after PSM treatment. Multivariate logistic regression analysis showed that VE (*p* = 0.015, OR = 1.750, 95% CI = 1.115 to 2.746, *E*-value = 2.90) and SA (*p* < 0.001, OR = 1.052, 95% CI = 1.030 to 1.074, *E*-value = 1.29) were the predictors of IA. The model successfully classified 81.7% of cases overall (*R*^2^_N_ = 0.133). Further analysis indicated that SA mediates between VE and IA (*Z* = 3.644, *p* < 0.001).

**Conclusion:**

This study revealed the potential mediation effect of SA on the link between VE and IA. By acknowledging the mediating influence of SA, researchers and practitioners can develop more accurate and effective strategies to mitigate Internet Addiction among adolescents.

## 1 Introduction

Internet users has grown exponentially to more than 5.8 billion worldwide by 2023 (Statista, 2023), making Internet Addiction (IA) receive increased attention from various stakeholders (e.g., the popular media, government authorities, and researchers) (Christakis and Moreno, 2009; Sun et al., 2019). Internet Addiction, also known as Internet Addiction Disorder (IAD) or Problematic Internet Usage (PIU), is commonly described as the problematic and compulsive utilization of the internet, resulting in clinically significant impairment or distress (Shaw and Black, 2008). IA is characterized by withdrawal, poor planning abilities, tolerance, preoccupation, impairment of control, and excessive online time, according to the diagnostic criteria proposed by Young (2009). One kind of Internet Addiction “Internet Gaming Disorder (IGD)” has been included in the 5th edition of The Diagnostic and Statistical Manual of Mental Disorders (DSM-5) (American Psychiatric Association, 2013). A systematic review and meta-analysis including 113 epidemiologic studies over 31 nations reported the global prevalence of Internet Addiction was 7.02% (95% CI, 6.09–8.08%) (Pan et al., 2020), and some studies have indicated young people are at particular risk of developing Internet Addiction (Lozano-Blasco et al., 2022). IA has been acknowledged as a significant public health concern due to its potential to induce various adverse effects on individuals, particularly teenagers, including but not limited to inattention, depression, social anxiety, loneliness, and a range of cognitive, emotional, and behavioral complications (Pan et al., 2020).

Internet Addiction, classified as a form of behavioral addiction, is influenced by various factors such as personal characteristics, online behaviors, parenting styles, familial dynamics, family and school environments, peer relationships, and psychological factors like loneliness and social anxiety (Weinstein and Lejoyeux, 2010; Xu et al., 2012; Li et al., 2019; Nielsen et al., 2020; Bu et al., 2021; Chemnad et al., 2023). Among the theoretical frameworks employed to understand this phenomenon, Agnew’s General Strain Theory (GST) stands out, which is a notable theoretical framework utilized to comprehend various behavioral issues, initially conceived to elucidate delinquency (Agnew, 1985; Agnew and White, 1992). GST focuses on the role of strain or stress in individuals’ lives, positing that strain arises when individuals perceive a disjunction between their desired goals and their ability to achieve them, or when they experience the removal of positively valued stimuli (Agnew and White, 1992). Over time, GST has been applied to understand a range of deviant behaviors, including alcohol abuse (Swatt et al., 2007), excessive drug use (Zweig et al., 2015) and problematic internet use among adolescents (Shi and Wang, 2023). Moreover, the GST framework has been utilized to investigate the association between offline bully-victimization and cyberbullying behavior (Jang et al., 2014), as well as between relational victimization and video game addiction among young individuals (Niu et al., 2022). According to GST, individuals encounter various stressors (e.g., victimization experiences, VE) that disrupt their overall state of well-being and trigger emotional responses, including anger, frustration, or anxiety (Agnew, 1985; Agnew and White, 1992). Victimization encompasses multiple dimensions, and some scholars consider physical, verbal, relational and cyberbullying victimization as the main forms (Raskauskas and Stoltz, 2007; Li et al., 2021). When faced with stress, adolescents may resort to maladaptive behaviors, such as excessive substance abuse or problematic internet use, as a means of escaping distressing situations. Hence, GST serves as a theoretical foundation for understanding the association between VE and IA. Previous studies have suggested that multidimensional victimization (i.e., domestic violence and bullying victimization) may be associated with an increased likelihood of psychological symptoms and Internet Addiction in adolescents (Hsieh et al., 2016; Li et al., 2021). However, further research is warranted to unravel the underlying mechanisms driving this association. Social Anxiety (SA), a significant psychological factor within the realm of IA, can be conceptualized as a reaction to the strain induced by experiences of victimization. Those grappling with social anxiety often encounter obstacles in establishing and maintaining positive interpersonal connections in offline settings, which can lead to additional maladaptive behaviors such as excessive internet usage (Shin and Newman, 2019). Consequently, individuals afflicted with social anxiety may exhibit a preference for engaging in online interactions, as the perceived anonymity and diminished face-to-face interaction afford them a heightened sense of security. Through the perspective of GST, the link between VE, SA, and IA is clearer. Stressful experiences (e.g., VE) lead to negative emotions (e.g., SA) and maladaptive behaviors, such as excessive internet use to cope with distress. However, this is only a theoretical proposition and deserves empirical investigation. Although previous studies have shown that victimization experiences are positively associated with depression, anxiety, and Internet Addiction (Li et al., 2021), it remains uncertain whether social anxiety plays a mediating role between adolescent victimization experiences and Internet Addiction in real-world settings.

Therefore, attempting to fill in this gap, we performed a large cross-sectional survey in 11 middle schools or high schools (a total of 78 high school) in Macao (Special Administrative Region of China) with a population of 680,000. This study aimed to examine the mediating role of SA in the relationship between VE and IA among adolescents. To mitigate potential biases in observational data, a Propensity Score Matching (PSM) technique used in some previous mediation analysis research (Jo et al., 2011; Yang et al., 2022) was employed in the present study. The findings will provide a more comprehensive understanding of the underlying mechanisms driving IA, and a novel insight for prevention and intervention strategies.

## 2 Methods

### 2.1 Participants and procedure

A cross-sectional survey using a structured questionnaire was conducted among 11 middle schools or high schools in Macao. Inclusion criteria were students from Form 2 to Form 6 who were able to answer Chinese e-questionnaires online. The minimum sample size was around 1,067 based on the sampling formula $n=\frac{Z_{\alpha /2}^{2}p\left(1-p\right)}{\delta^{2}}$ (*α* = 0.05, δ =0.03, *p* = 0.5) (Cochran, 1977). A multistage cluster sampling approach was used to ensure representativeness and minimize bias. Firstly, 10 schools were purposely selected in different districts of Macao according to their students’ number (large, medium or small). Secondly, a sample of 2–4 classes was chosen from each school, and all students in the selected classes were invited to take part. Thirdly, an extra school was invited to participate to supplement the sample size. The cross-sectional study was conducted by an online questionnaire platform. Participants willingly volunteered to partake in the survey and provided written informed consent. The survey was conducted between October and December 2022, following a pilot study.

### 2.2 Measures

The self-administered questionnaire included (i) Internet Addiction, (ii) Social Anxiety, (iii) Victimization, and (iv) Covariates.

#### 2.2.1 Internet Addiction (IA)

IA was measured with the eight-item Diagnostic Questionnaire (DQ) for Addictive Internet use revised by Young (2009). Respondents who answered “yes” to five or more of the criteria were classified as addicted Internet users and the remainder were classified as normal Internet users. The measurement is used worldwide and was adopted to measure Internet Addiction among adolescents in Macao before (Government of Macao Special Administrative Region Social Welfare Bureau, 2012). The Cronbach’s α coefficient of the scale was 0.81 in the present study with sufficient reliability.

#### 2.2.2 Social anxiety (SA)

SA was measured with the Social Anxiety Scale for Adolescents (SAS-A), which was developed to measure social anxiety symptoms (La Greca and Lopez, 1998). In this study, we used a shortened version containing 12-items (Nelemans et al., 2019). Each item of the scale was rated on 5-point Likert scale from 1 (not at all) to 5 (all the time) in response to statements about how much the description was “true for you.” The SAS-A included three subscales: Fear of Negative Evaluation (SAS-A-FNE) (Items 1–4), Social Avoidance and Distress in the new social situation (SAS-A-New) (Items 5–8), and Social Avoidance and Distress in general (SAS-A-G) (Items 9–12). The total score ranged from 12 to 60, a higher score indicating a higher Social Anxiety. Previous study has proven that the shortened version of SAS-A has good reliability and validity among Chinese spoken teenagers (Sun et al., 2017). And the Cronbach’s α coefficient of SAS-A was 0.93 in the present study with sufficient reliability.

#### 2.2.3 Victimization experiences (VE)

In accordance with prior research (Glüer and Lohaus, 2015; Stewart-Tufescu et al., 2021), three filter questions were employed for the assessment of VE. These questions were “Have you experienced domestic physical victimization in the past year?”, “Have you experienced domestic verbal victimization in the past year?”, “Have you experienced cyberbullying in the past year?”. If the respondents answer no to all questions, it will be defined as non-victimized; others were defined as victimized.

#### 2.2.4 Covariates

These consisted of gender, age, grade, living with siblings or not, having bad habits (smoking, drinking, gambling, etc.) or not, and the Junior Students’ Internet Literacy (JILS). Previous studies found family factors, such as interaction and quality of the parent–child relationship, significantly impact adolescents’ Internet Addiction (Pinquart, 2017; Shek et al., 2019; Cai et al., 2021), so degree of enjoy communication with family (DECF) and degree of enjoy family activities (DEFA) were also included. DECF and DEFA were rated with a 5-point Likert-type scale (1 = dislike very much, 5 = like very much). JILS containing 18 items were rated on a 5-point Likert scale (1 = strongly disagree, 5 = strongly agree), a higher score indicating a higher internet literacy (Huang et al., 2021). Cronbach’s *α* coefficient of JILS was 0.84 in the present study with sufficient reliability.

### 2.3 Statistical analysis

A propensity score matching method was conducted in this study. A propensity score of each participant was predicted using multivariable logistic regression, and 1:1 matching of the scores in the groups of victimized or not was performed (Thoemmes, 2012). The match tolerance was set at 0.02. The following covariates were used for the PSM analysis: gender, age, grade, living with siblings, having bad habits, DECF, DEFA and JILS. A balance-check of the covariates was performed to ascertain the appropriateness of employing this method, utilizing both *p*-value and standardized mean difference (SMD) as evaluation measures.

Additionally, the following four steps proposed by Baron and Kenny (1986) were conducted to establish mediation effect. Step1: the variable of VE significantly affects the variable of IA in the absence of SA, Step2: the variable of VE significantly affects the variable of SA, Step3: SA has a significant unique effect on IA, and Step4: the effect of VE on IA shrinks upon the addition of SA to the model. In present study, least squares linear regression analysis was used in Step2 and logistic regression analysis was used in Step 1, 3 and 4. All results of regression analyses are presented in regression coefficients (*β*) and related standard errors (SE). Because least squares linear regression coefficients are differently scaled with logistic regression coefficients, we used the Sobel test for categorical outcome variables proposed by Iacobucci (2012) to check the significance of mediation in Step 4. And calculation for the Sobel test can be conducted online.^1^ Lastly, like all analytical approaches utilizing observational data, including propensity score methods, residual biases will persist, and only measurable variables can be addressed. Thus, the *E*-value, denoting the minimal level of correlation required between an unobserved confounding variable and both the treatment and outcome variables to effectively account for unmeasured confounding (VanderWeele and Ding, 2017), was computed utilizing a publicly accessible online calculator.^2^ Data were analyzed with SPSS software (Version 28). In all calculations, significance was accepted at the *p*-value <0.05.

## 3 Results

### 3.1 Participant characteristics

**Figure 1:**
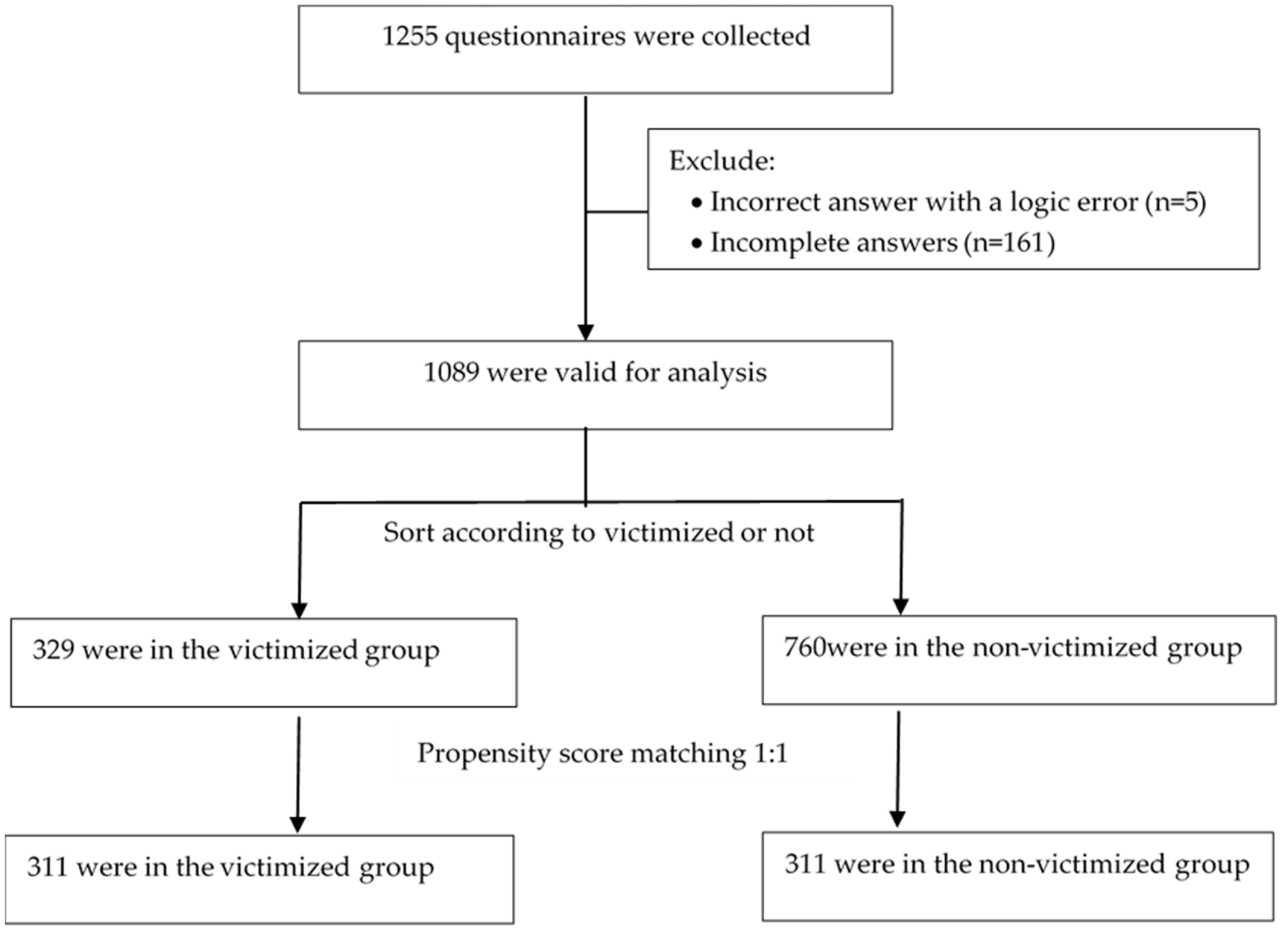
Flowchart of data processing.

A total of 1,255 questionnaires were collected and 1,089 (86.8%) were valid for analysis. As shown in Table 1, their age ranged from 11 to 25(mean = 15.41, SD = 1.82), 69.8% the participants had experienced domestic physical or verbal victimization or cyberbullying in the past year. And 20.6% of the participants were considered to have Internet Addiction. Figure 1 illustrates that initially, 329 participants were allocated to the victimized group, while 760 were assigned to the non-victimized group. Subsequently, 311 pairs were selected through PSM treatment based on the criteria outlined above.

**Table 1:** Participant characteristics (*n* = 1,089).

Variables	Count	%	Mean ± SD
Gender			
Male	553	50.8	
Female	536	49.2	
Grade			
Form 2	237	21.8	
Form 3	170	15.6	
Form 4	283	26.0	
Form 5	178	16.3	
Form 6	221	20.3	
Living with siblings			
No	214	19.7	
Yes	875	80.3	
Major setbacks experience			
No	345	31.7	
Yes	744	68.3	
Victimization experiences			
No	329	30.2	
Yes	760	69.8	
Internet addiction			
No	865	79.4	
Yes	224	20.6	
Age			15.41 ± 1.82
DECF			3.25 ± 1.068
DEFA			3.06 ± 1.099
JILS			71.82 ± 9.16
SA			36.43 ± 11.52

JILS, Junior Students’ Internet Literacy; SA, Social Anxiety. DECF, degree of enjoy communication with family; DEFA, degree of enjoy family activities.

### 3.2 Balance-check of the covariates before and after PSM

Table 2 shows the comparisons of baseline characteristics before and after PSM treatment. Before PSM, there were significant differences (*p* < 0.05; SMD > 0.10) among bad habits, degree of enjoy communication with family (DECF) and degree of enjoy family activities (DEFA) between the victimized and the non-victimized groups. However, there were no significant differences between the two groups regarding the above-mentioned characteristics after PSM. Additionally, respondents in the victimized group reported significantly higher Internet Addiction than those in non-victimized group (23.5% vs. 12.5%, *p* < 0.001) after PSM.

**Table 2:** PSM to balance the participants’ characteristics between the victimized and non- victimized groups.

Characteristics	Before PSM				After PSM			
	Victimized group	Non-victimized group	SMD	*p*-value	Victimized group	Non-victimized group	SMD	*p*-value
No. of participants	760	329			311	311		
Age, mean ± SD	15.35 ± 1.80	15.55 ± 1.87	0.11	0.097	15.44 ± 1.84	15.47 ± 1.83	0.02	0.709
JILS, mean ± SD	71.57 ± 8.96	72.39 ± 9.60	0.09	0.174	72.32 ± 9.33	72.20 ± 9.72	0.01	0.879
DECF, mean ± SD	3.09 ± 1.043	3.61 ± 1.039	0.50	<0.001	3.56 ± 1.01	3.56 ± 1.03	0.00	0.987
DEFA, mean ± SD	2.90 ± 1.059	3.43 ± 1.102	0.49	<0.001	3.37 ± 1.11	3.36 ± 1.09	0.01	0.537
Gender			0.06	0.066			0.01	0.747
Male	372 (48.9%)	181 (55.0%)			171 (55.0%)	167 (53.7%)		
Female	388 (51.1%)	148 (45.0%)			140 (45.0%)	144 (46.3%)		
Grade			0.08	0.119			0.04	0.883
Form 2	170 (22.4%)	67 (20.4%)			67 (21.5%)	67 (21.5%)		
Form 3	120 (15.8%)	50 (15.2%)			48 (15.4%)	47 (15.1%)		
Form 4	210 (27.6%)	73 (22.2%)			81 (26.0%)	71 (22.8%)		
Form 5	118 (15.5%)	60 (18.2%)			53 (17.0%)	58 (18.6%)		
Form 6	142 (18.7%)	79 (24.0%)			62 (19.9%)	68 (21.9%)		
Living with siblings			<0.01	0.954			0.03	0.416
No	149 (19.6%)	65 (19.8%)			56 (18.0%)	64 (20.6%)		
Yes	611 (80.4%)	264 (80.2%)			255 (82.0%)	247 (79.4)		
Bad habits			0.09	0.005			0.04	0.307
No	703 (92.5%)	319 (97.0%)			296 (95.2%)	301 (96.8%)		
Yes	57 (7.5%)	10 (3.0%)			15 (4.8%)	10 (3.2%)		
Internet addiction			0.13	<0.001			0.14	<0.001
No	578 (76.1%)	287 (87.2%)			238 (76.5%)	272 (87.5%)		
Yes	182 (23.9%)	42 (12.8%)			73 (23.5%)	39 (12.5%)		

JILS, Junior Students’ Internet Literacy; SA, Social Anxiety. DECF, degree of enjoy communication with family; DEFA, degree of enjoy family activities.

### 3.3 The mediation effect of SA between VE and IA

Table 3 shows that Victimization Experiences (*p* = 0.015, OR = 1.750, 95% CI = 1.115 to 2.746) and Social Anxiety (*p* < 0.001, OR = 1.052, 95% CI = 1.030 to 1.074) were the significant independent predictors of Internet Addiction. The model successfully classified 81.7% of cases overall (*R*^2^_N_ = 0.133). In the sensitivity analysis, the E-value for the OR of 1.750 was 2.90 and for the OR of 1.052 was 1.29. It means the OR for VE and SA could be explained completely by residual confounding if an unmeasured confounder had an Odds Ratio association above 2.90 and 1.29 with Internet Addiction. Furthermore, the mediation effect analysis in Table 4 shows that Social Anxiety Mediates between Victimization Experiences and Internet Addiction (*Z* = 3.644, *p* < 0.001).

**Table 3:** The logistic regression analysis for internet addiction (*n* = 622).

Variables	*β*	S.E.	*Χ* ^2^	*p*-value	OR	95% C.I. for OR	
						Lower	Upper
Grade			4.261	0.372			
Grade (Form 3 = 1)	0.221	0.364	0.369	0.544	1.248	0.611	2.549
Grade (Form 4 = 1)	−0.48	0.395	1.472	0.225	0.619	0.285	1.343
Grade (Form 5 = 1)	0.018	0.457	0.002	0.969	1.018	0.415	2.495
Grade (Form 6 = 1)	−0.289	0.514	0.317	0.574	0.749	0.273	2.051
Age	0.100	0.097	1.064	0.302	1.105	0.914	1.336
Gender (Female = 1)	−0.183	0.227	0.654	0.419	0.832	0.534	1.298
Living with siblings (Yes = 1)	−0.236	0.274	0.741	0.389	0.79	0.461	1.352
Bad habits (Yes = 1)	0.272	0.505	0.289	0.591	1.312	0.488	3.530
DEFA	0.112	0.146	0.594	0.441	1.119	0.841	1.488
DECF	−0.232	0.158	2.158	0.142	0.793	0.582	1.081
JILS	−0.020	0.012	2.726	0.099	0.980	0.957	1.004
VE	0.560	0.230	5.926	0.015	1.750	1.115	2.746
SA	0.051	0.011	23.048	<0.001	1.052	1.030	1.074
Constant	−2.991	1.665	3.225	0.073	0.050		

JILS, Junior Students’ Internet Literacy; VE, Victimization Experiences; SA, Social Anxiety. DECF, degree of enjoy communication with family; DEFA, degree of enjoy family activities.

**Table 4:** Mediation effect of social anxiety between victimization and internet addiction (*n* = 622).

Analysis	*β*	S.E.	*Χ*^2^/t	*p*-value
Step 1	0.792	0.221	12.831	<0.001
Step 2	5.364	0.910	5.896	<0.001
Step 3	0.051	0.011	23.048	<0.001
Step 4	0.560	0.230	5.926	0.015
Sobel test	*Z* = 3.644, *p* = <0.001			

The models are adjusted for gender, age, grade, living with siblings, bad habits, DEFA, DECF, and JILS.

## 4 Discussion

The present study delved into the intricate relationship between victimization experiences, social anxiety, and internet addiction among adolescents. Through the application of PSM, we sought to uncover the potential mediation effect of social anxiety on the link between victimization experiences and internet addiction. The results indicated that the experience of victimizations was positively associated with adolescents’ internet addiction through the mediating effects of social anxiety.

### 4.1 The impact of VE on IA

The results presented in Table 2 demonstrate that individuals in the victimized group reported a significantly higher prevalence of Internet Addiction compared to those in the non-victimized group (23.5% vs. 12.5%) after employing PSM. Furthermore, the logistic regression analysis presented in Table 3 supports a positive association between Victimization Experiences and Internet Addiction. These findings from the current study contribute substantial evidence to the understanding of the noteworthy influence of victimization experiences on the emergence of internet addiction among adolescents. Moreover, these results align with prior research that has emphasized the detrimental consequences of victimization experiences. For instance, a study conducted in Taiwan surveyed a nationally representative sample of 6,233 fourth-grade primary school students. The findings revealed a positive correlation between participants’ multidimensional victimization experiences and the development of psychological symptoms and internet addiction. Similarly, a study conducted in Turkey examined 2,422 voluntary high school students and found that cyber victimization and cyberbullying were associated with specific Internet usage characteristics and Internet Addiction (Simsek et al., 2019). Another survey of 2,843 secondary students in China also found that experience of victimization was positively associated with depression and anxiety, as well as Internet Addiction (Li et al., 2021). Some other studies also found similar results (Lin et al., 2020; Cao et al., 2021). However, Internet Addiction is linked to various factors, including personality traits, parenting styles, and familial influences (Weinstein and Lejoyeux, 2010; Zhang et al., 2018; Nielsen et al., 2020; Chemnad et al., 2023). The present study illustrated that people who have different VE may also have significant differences in terms of having bad habits (smoking, drinking, gambling, etc.), DECF and DEFA before PSM (Table 2). These factors can introduce confounding biases, which previous studies often failed to exclude as alternative explanations. In contrast, the present study mitigated these confounding biases through the utilization of PSM methodology. Previous study demonstrated that PSM is overall a more favorable approach than traditional regression analysis when estimating causal effects using observational data. Thus, this study contributes valuable evidence to the existing body of research, as it further expands upon the topic by specifically illustrating that adolescents who have experienced victimization are at a heightened risk of developing Internet Addiction.

### 4.2 The mediation effect of SA between VE and IA

A significant contribution of this study lies in its identification of social anxiety as a potential mediator in the relationship between victimization experiences and internet addiction among adolescents. This phenomenon can be explained with Agnew’s General Strain Theory, as the experience of victimization may give rise to adverse emotional states, including social anxiety. And individuals grappling with social anxiety encounter challenges in establishing and sustaining positive interpersonal connections in the offline realm, potentially resulting in additional maladaptive behaviors such as excessive internet usage. Several previous studies have explored the mechanism between victimization experiences and Problematic Internet Usage. For instance, a study conducted in China during the COVID-19 pandemic found that social anxiety acts as a mediator between relational victimization and video game addiction among female college students (Niu et al., 2022). Additionally, another study demonstrated that psychological morbidity, such as depression and anxiety, serves as a mediating factor in the influence of victimization on problematic online behavior, including Internet Addiction and cyberbullying (Li et al., 2021). These findings exhibit partial inconsistency with the current study, yet demonstrate a degree of concurrence. Consequently, it can be inferred that there exist various mechanisms linking Victimization Experiences and Internet Addiction, with Social Anxiety representing merely one facet among them (the result of Step 4 analysis in Table 4 also supports this point). From a GST perspective, experiences of victimization result in strain, which subsequently leads to an increase in social anxiety as a coping mechanism. This heightened social anxiety may then prompt individuals to excessively use the internet as a means of avoiding face-to-face social interactions and alleviating distress (Feng et al., 2019). Consequently, these findings contribute to the existing body of research and offer a more comprehensive comprehension of the underlying mechanisms that contribute to IA. Additionally, these findings offer novel insights for interventions and prevention strategies targeting IA among adolescents.

### 4.3 Implications

The results of this study have significant practical implications for stakeholders involved in the well-being of adolescents in the digital age. Specifically, recognizing social anxiety as a mediating factor highlights the need to address not only the direct experience of victimization, but also its emotional consequences. Firstly, school-based interventions and counseling programs should consider the potential psychological consequences of victimization, with particular attention to the increased likelihood of developing social anxiety. A review examined a number of strategies for addressing anxiety in children and adolescents, including social interaction, contact with nature, sensory stimulation, physical activity, etc. (Wolpert et al., 2019). Previous research has also highlighted the importance of resilience-based intervention strategies in mitigating the risk of anxiety associated with bullying and cyberbullying victimization (Hinduja and Patchin, 2017; Fang et al., 2022). Therefore, it is recommended that programs that develop emotional resilience and coping mechanisms, such as stress management and prosocialness training (i.e., the willingness to assist, help, share, care and empathy with others) (Diotaiuti et al., 2021b), could be included in the curriculum. Secondly, parents are crucial in mitigating the adverse effects of victimization experiences and internet addiction on adolescents. Parents can create a safe space for their children by being aware of the potential consequences of victimization and social anxiety (Özdemir, 2014). Therefore, education for parents regarding this should be strengthened. Finally, policymakers can use research findings to advocate for policies that effectively address youth experiences of victimization in both online and offline domains. And appropriate allocating resources toward school-based counseling services for children and families can significantly contribute to the prevention and management of IA associated with SA (Schalkwyk and Sit, 2013). Moreover, it is important to promote a sense of safety and empower adolescents to control their environment and relationships, and increase their sense of control can help restore confidence in their ability to protect themselves quickly after victimization (Diotaiuti et al., 2021a).

### 4.4 Limitations and future research

Several limitations should be acknowledged when interpreting the findings. First, although the PSM method is generally consistent with randomized clinical trials when applied appropriately (Ross et al., 2015), it possesses its own limitations because it may result in the exclusion of observations without suitable matches, leading to potential loss of data information in the final estimation. Second, although the study provides insights into the mediating role of SA, it is crucial to recognize the possibility of bidirectional relationships and the presence of other mediating factors that may impact the pathway between VE and IA. Furthermore, the utilization of a cross-sectional design in this study imposes limitations on the capacity to establish causality. Additionally, the collection of VE relied on participant recall rather than objective records and we had to admit that stressor from peers was not included in the study which may lead to an underestimation of victimization. Moreover, it is important to note that the case of Macao, being an Asian city with the highest population density globally, may not possess high representativeness on a global scale. Therefore, caution must be exercised when interpreting and generalizing the findings.

In future research, it is recommended to conduct some longitudinal studies, as they can reveal the time series of variables and contribute to a more comprehensive understanding of the underlying dynamics. Additionally, it is interesting to investigate the boundary conditions of the mechanism linking SA, VE, and IA, because of a prior study’s findings, which indicated that anxiety mediated the association between VE and IA in boys but not in girls (Li et al., 2021). Finally, it would be beneficial to conduct intervention studies specifically aimed at addressing SA among students who have experienced victimization, and further demonstrate the application value of this research’s findings.

## 5 Conclusion

In conclusion, this research adds to the expanding corpus of scholarly work investigating the complex relationship between victimization experiences, social anxiety, and internet addiction in the adolescent demographic. Utilizing the PSM method to control possible confounding bias, we revealed the potential mediation effect of social anxiety on the link between victimization experiences and internet addiction. By acknowledging the mediating influence of social anxiety, researchers and practitioners can develop more accurate and effective strategies to mitigate Internet Addiction among adolescents.

## Data availability statement

The raw data supporting the conclusions of this article will be made available by the authors, without undue reservation.

## Ethics statement

The studies involving humans were approved by Kiang Wu Nursing College of Macau (Reference number: 2021OCT01) and the Science and Technology Development Fund of Macao Special Administrative Region (Reference number: 0024/2021/ITP). The studies were conducted in accordance with the local legislation and institutional requirements. Written informed consent for participation in this study was provided by the participants' legal guardians/next of kin.

## Author contributions

JW: Data curation, Methodology, Writing – original draft. HW: Conceptualization, Funding acquisition, Methodology, Supervision, Writing – review & editing. XL: Data curation, Investigation, Writing – review & editing. IV: Investigation, Writing – review & editing. XX: Writing – review & editing. CP: Writing – review & editing.

## Footnotes

^1^ https://quantpsy.org/sobel/sobel.htm

^2^ https://www.evalue-calculator.com/
